# Expansion of a Novel Subset of PD1+CXCR5-CD4+ T Peripheral Helper Cells in IgG4-Related Disease

**DOI:** 10.14309/ctg.0000000000000111

**Published:** 2020-01-21

**Authors:** Tamsin Cargill, Eleanor Barnes, Emma L. Culver

**Affiliations:** 1 Oxford NIHR BRC and Peter Medawar Building for Pathogen Research, Nuffield Department of Medicine, University of Oxford, Oxford, UK;; 2 Translational Gastroenterology Unit, John Radcliffe Hospital, Oxford, UK.

We thank Akiyama et al. (1) for providing evidence in support of our data demonstrating the importance of circulating-activated (PD1+CXCR5+CD4+) and tissue-resident (CXCR5+CD4+) T follicular helper (Tfh) cells in patients with IgG4-related disease (IgG4-RD) and the role of Tfh cells supporting B cells to differentiate into plasmablasts to produce IgG4 antibodies (2). They highlight important unresolved issues, specifically drivers for Tfh cell expansion in patients with IgG4-RD.

We wish to highlight the presence of a unique subset of circulating T peripheral helper (Tph PD1^hi^CXCR5-CD4+) cells in IgG4-RD, which may be able to recruit both Tfh cells and B cells to the sites of inflammation. Pathologically expanded Tph cells were recently identified in the circulation and nonlymphoid rheumatoid joints in patients with rheumatoid arthritis (3), and expressed factors including IL21, CXCL13, and inducible co-stimulatory molecule, enabling B cell help. Here, we show for the first time an increase in the percentage of circulating PD1^hi^CXCR5-CD4+ Tph cells in patients with active IgG4-related pancreatic and biliary disease compared with treated inactive disease (*P* = 0.002) and healthy volunteers (*P* = 0.04) (Figure 1, top panels). The percentage of Tph cells correlates with the serum IgG4 level (*P* = 0.02, r0.75) and IgG4-responder disease activity index (*P* = 0.003, r = 0.71) (Figure 1, bottom panels). The absolute number of circulating Tph cells falls with corticosteroid treatment (*P* = 0.03). We have demonstrated coexpression of CD4+ T cells with CXCL13, a ligand of CXCR5, and inducible co-stimulatory molecule in affected tissues (pancreas, bile duct, and salivary gland) in IgG4-RD (2).

**Figure 1.**
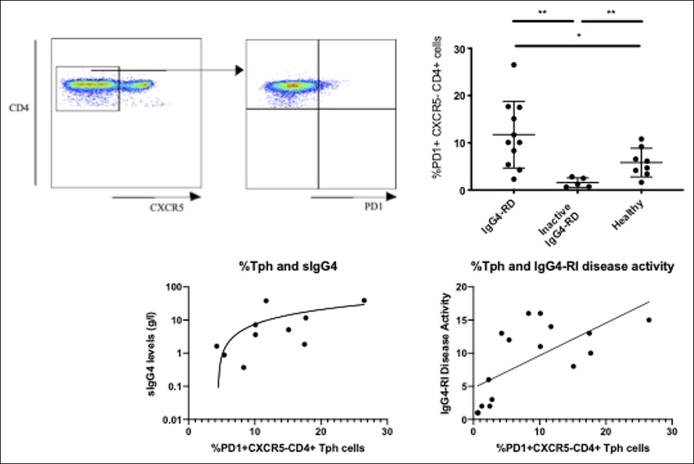
Circulating Tph cells in IgG4-RD. Top left panel shows gating strategy to identify PD1+CXCR5-CD4+ Tph cells. Top Right panel shows Tph cells (PD1+) as a percentage of CXCR5- CD4+CD45RA cells in patients with active IgG4-RD (n = 11), inactive IgG4-RD (n = 5), and healthy controls (n = 9). Median and interquartile range shown. Mann–Whitney nonparametric test and Kruskal–Wallis test with Dunn correction for multiple comparisons between groups *P < 0.05; **P < 0.01. Bottom Left panel shows the spearman rank correlation between serum IgG4 (log10) and percentage of Tph cells in IgG4-RD (*P* = 0.02; r = 0.75). Bottom Right panel shows the spearman rank correlation between IgG4-responder index disease activity score and percentage of Tph cells in IgG4-RD (*P* = 0.003; r = 0.71). IgG4-RD, IgG4-related disease; Tph, T peripheral helper.

Expanded Tph cells can express chemokine receptors that direct migration to inflamed sites, such as CCR2 and CCR5, and chemokines such as CXCL13, which may recruit CXCR5-expressing immune cells, including Tfh cells and B cells to initiate and maintain inflammation (4). As such, Tph cells may play a more pathogenic role than their Tfh counterparts in IgG4-RD immune pathogenesis. Key questions remain in the developmental relationship, origin, and differentiation of such cells in the context of IgG4-RD. Understanding the mechanisms underlying Tfh and Tph cell-mediated immunity and pathology may bring potential targets for novel therapies in this disease.

## CONFLICTS OF INTEREST

**Guarantor of the article:** Emma L. Culver, BSc, MBChB, MRCP, DPhil(Oxon).

**Specific author contributions:** T.C. recruited patients, collected samples, assessed disease activity, performed the Tph cell flow cytometry, analyzed data. E.B. is the principle investigator for the IgG4-RD cohort study. E.L.C. had the original concept for this study, drafted the manuscript, recruited patients, assessed disease activity and serology levels, performed flow assays, and performed data analysis/interpretation. All authors edited and approved the final manuscript.

**Financial support:** NIHR and BRC Oxford. This study was supported by the National Institute of Health Research (NIHR) Biomedical Research Centre, based at Oxford University Hospitals Trust and Oxfordshire Health Service Research Committee (OHSRC) as part of Oxford Hospitals Charity, Oxford. The views expressed in this article are those of the authors and not necessarily those of the NHS, the NIHR, or the Department of Health.

**Potential competing interests:** E.L.C. consults for Xencor and Viela Bio for IgG4-related disease.
